# Functional Hydrogel-Based Flexible Thermoelectric Generators: Principles, Mechanism, and Emerging Applications

**DOI:** 10.3390/gels12070598

**Published:** 2026-07-03

**Authors:** Md Murshed Bhuyan, Jae-Ho Jeong

**Affiliations:** 1 Department of Mechanical, Smart, and Industrial Engineering (Mechanical Engineering Major), Gachon University, 1342, Seongnam-daero, Sujeong-gu, Seongnam-si 13120, Republic of Korea; mdmurshed86@gachon.ac.kr; 2 School of Mechanical Engineering, College of Engineering, Chung-Ang University, 84 Heukseok-ro, Dongjak-gu, Seoul 06974, Republic of Korea

**Keywords:** gels/hydrogel, thermoelectric generator, thermodiffusion, Seebeck coefficient, conductivity

## Abstract

One of the latest and innovative areas of research in energy is the development of thermoelectric generators (TEGs). A novel family of soft, sustainable energy harvesters, hydrogel-based renewable flexible thermoelectric generators use linked ionic, electronic, and redox processes to transform heat gradients into electrical energy. According to recent research, a hydrogel-based TEG has ionic Seebeck coefficients (S) of the order 10–40 mV K^−1^, which are tens to hundreds of times greater than those of electronic polymers. Thermal conductivities are modest (~0.3–0.6 W/m·K), ionic conductivities typically vary from 10^−3^ to 10^−1^ S cm^−1^, and water-rich gels are naturally soft with elastic moduli ~10^3^–10^6^ Pa and elongations > 100–800%. Recent developments in the concepts, properties, working mechanism, and potential applications of hydrogel-based thermoelectric generators are the focus of this review paper. We investigate the basic transport processes, such as ionic thermodiffusion, thermoelectric ion–electron coupling, and redox-mediated potential production, that allow thermoelectric conversion in hydrogels. This review identifies bottlenecks such as poor output power under minor gradients, summarize performance parameters, and assess methods to improve efficiency. Wearable and implanted power sources, low-grade waste heat collection, and environmental monitoring are examples of promising applications. Lastly, we describe the research avenues that must be pursued in order to expedite the transition of hydrogel-based thermoelectric generators from lab tests to useful, sustainable energy sources. Therefore, the review can provide fundamental knowledge on hydrogel-based TEGs along with their working principles.

## 1. Introduction

Nowadays, transportation, the chemical industry, metallurgical and other fields are significant waste heat resources, with the majority of waste heat temperature falling between 400 and 800 °C [1,2]. The production of electricity from natural gas, fuel, and coal harms people due to their high capacity for atmospheric pollution and global warming. The US Energy Information Administration (EIA) reported that electricity generation from power plants gradually increased yearly by about 28% in 2014, 35% in 2018, and 39% in 2019 [3]. Additionally, liquid fuel consumption and production rose by about 6 million barrels daily from mid-2014 to mid-2018, indicating the enhanced energy cost [4]. To solve this problem, scientists emphasize discovering sustainable and environmentally friendly energies. Recently, scientists have focused on renewable energy due to its available sources like RF radiation, thermal, solar, mechanical research, etc., transforming it into electrical energy. Managing energy in the current world is very important due to industrialization and population growth. Researchers are utilizing different energy sources like sunlight, wind, and nuclear power as energy sources and storing them safely and efficiently.

Therefore, research on thermoelectric generator technology based on locations with medium and feverish temperatures is very valuable. Thermoelectric (TE) generator (TEG) technology is becoming popular as it uses the Seebeck effect to convert thermal energy into electrical energy. It is considered a straightforward and sustainable way to utilize waste heat and decrease energy consumption [5]. PbTe [6], skutterudite [7,8], and half-Heusler (hH) alloy [9,10] are the most researched thermoelectric materials for medium- and high-temperature applications; they show excellent thermoelectric properties in the relevant temperature range. Skutterudite is affected by low thermal stability, while PbTe has the drawbacks of toxicity and poor mechanical qualities for large-scale industrial applications [11]. Interestingly, the compounds of group IV-VI of the periodic table like SnSe [12,13] have become a crucial component with promising TE performance. To improve the energy-harvesting efficiency of TEGs, several cutting-edge techniques have been used, including the use of 3D charge and 2D phonon methods [4], band modification [5], multiband alignment [6], phonon–electron decoupling [7], and lattice planification [14]. Notably, the well-researched high-performance TE material PbTe [15,16] is a reliable benchmark for investigating quantum phenomena related to TE behavior, providing a solid foundation for our research. Notwithstanding their effectiveness, SnSe and PbTe have low electrical conductivity at temperatures close to room temperature, mainly caused by their comparatively large bandgap numbers. Because of this feature, they cannot capture heat energy at lower temperatures, where a significant amount of wasted heat is usually present. TEG works by exploiting the temperature gradient between two individual materials. Typically, one side is heated while the other side remains cooler. Thermoelectric power has no moving parts and is compact, dependable, and environmentally friendly. As a result, the scientist takes it seriously for further exploration [15,17,18,19,20].

Thermoelectric generators (TEGs) are increasingly acknowledged for their potential to convert waste heat into electricity, offering a sustainable energy solution across various applications. Their versatility spans automotive, industrial, and residential sectors, enhancing energy efficiency and reducing emissions. TEGs can recover waste heat from vehicle exhaust systems, significantly increasing the energy efficiency in automotive applications [21]. The design and selection of TEGs are important, particularly in low-temperature environments (below 500 K), where they outperform traditional power generation methods [22]. Thermoelectric generators (TEGs) are considered promising technologies for energy conversion, but several limitations prevent their widespread application. These limitations can be categorized into efficiency, cost, and design constraints. First, TEGs typically exhibit low efficiency, ranging from 2.5% to 6.5% in various applications [23]. The efficiency depends on the temperature gradient; for example, in vehicle exhaust applications, efficiencies can vary between 3.6% and 15.9% based on exhaust temperatures [24]. Secondly, the cost of TEG systems is significantly high, estimated between 2000 and 15,000 USD per kW [23]. This high expense limits their adoption, especially compared to other renewable technologies. Thirdly, geometric constraints arise from manufacturing processes, such as screen printing, which restricts layer thickness and device architecture [25]. Furthermore, the performance of TEGs is also influenced by the thermoelectric figure of merit (ZT), but it is not the sole determinant of system performance, as optimal configurations can vary [26]. Incorporation of gels/hydrogels can mitigate the limitations of TEGs, resulting in better efficiency and performance as well as flexibility.

Hydrogels are three-dimensional, crosslinked polymer networks that absorb bulk amounts of water, making them highly flexible materials in several fields, particularly biomedicine. Their unique properties, like biocompatibility and the ability to mimic natural tissue, enable applications ranging from drug delivery systems to tissue engineering. It contains hydrophilic polymer chains that swell upon contact with water, forming a gel-like substance [27]. They can be produced using different methods, including copolymerization and crosslinking of monomers, which can be tailored for specific applications [28,29].

A type of composite known as conductive hydrogels combines the mechanical attributes and drug-loading capability of hydrogels with the electrical functionality of conductive polymers. Because of their high hydrophilicity, crosslinked polymer networks are known as hydrogel stretch in water. Conductive hydrogels are considered promising materials for thermoelectric generators (TEGs) due to their unique properties, including high conductivity, flexibility, and moisture retention. It is widely used in wearable technology, where real-time energy is harvested from body heat [30]. Hydrogel dramatically increases the outcome of thermoelectric generators by providing a good cooling interface and serving as a component in triboelectric nanogenerators (TENGs) [31,32]. The ionic hydrogel can be incorporated to enhance the charge density at interfaces, leading to a rise in the output performance of TEGs and increasing the voltage and density of power [33]. The versatility of hydrogels prepares them for several applications, including the human–machine self-power interface, which indicates their capability for sustainable technologies [34]. Though hydrogels have the potential to improve performance, they have some challenges in optimizing their properties for application in TEGs [31]. Hydrogel pastes of the p- and n-type were created by Bo Wu et al. as printing inks for high-performance flexible thermoelectric generators (f-TEGs). By physically crosslinking the carboxylated cellulose nanofibers (CCNs) and entangling molecular chains, the pastes’ high viscosity was attained while also successfully reducing the fluidity of the nanorod dispersions. At a temperature differential of 70 K, an f-TEG with 70 TE couplings generated a voltage of around 500 mV, and at Δ*T* = 50 K, the specific power density reached 1.278 W m^−2^ [35]. In comparison to inorganic thermoelectrics, reported power densities and figures of merit for hydrogel-based TEGs are still low, and methods for optimizing thermopower, ionic conductivity, and mechanical compliance all at once are still lacking. For wearable applications, long-term stability, dehydration resistance, and mechanical durability under repetitive deformation and environmental exposure are not adequately described [36]. Also, scalable manufacturing, device encapsulation, and the electronics interface are examples of system-level integration issues that are rarely handled comprehensively [37,38]. Therefore, to allow medical and on-body deployment, more thorough research is needed on biocompatibility, safety, and performance across actual temperature gradients (low-grade heat harvesting). In order to speed up the creation of useful hydrogel thermoelectric generators, this study highlights these gaps, summarizes current developments, and suggests specific research avenues. This review paper analyses the current development, functional mechanism, obstacles, and opportunities in thermoelectric (TE) gels and hydrogels.

## 2. Polymer-Based Conductors and Their Applications

Conductive polymers (CPs) are a distinct type of material that combines the traditional properties of polymers with good-quality electric conductance and comparable conductivities to metals and semiconductors, σ = 10^2^ to 10^5^ S/cm [39]. Materials like polyanilines, polythiophenes, and polypyrrole operate through band gap theory, hopping, and tunneling to carry charges such as solitons and polarons, which can impact their conductance [40]. CPs are becoming popular in coatings to prevent corrosion and electromagnetic interference shielding, and are even popular in healthcare applications [41,42]. Due to their solid-like and liquid-like properties, they are suitable for a wide range of applications. By embedding electronically conductive polymer networks (such as PEDOT and PANI) into a water-swollen, crosslinked matrix, conductive polymers can be converted into hydrogels to produce soft, stretchable thermoelectric materials. This method allows wearable TEGs but necessitates careful control of electronic vs. ionic conduction, doping, and moisture/stability [43,44].

### 2.1. Organic Polymers as Thermoelectric Materials

The family of inherently conducting polymers and polymer composites with thermoelectric characteristics (Seebeck coefficient, electrical conductivity, thermal conductivity) are known as polymer thermoelectric materials. The performance of the device is determined by the material-level qualities of these basic building parts [45]. In Table 1, TE properties of some conjugative polymers are mentioned. To date, most of the work is carried out by using poly(3,4-ethylene dioxythiophene) (PEDOT) [46,47], although the highest power factors are obtained from the coordination polymer [48]. The electrical conductivity of the conjugated polymer can be enhanced by electrochemical or chemical doping that increases the charge carriers, such as polarons and di-polarons. However, doping can cause a decline, bringing the Fermi level closer to the conduction band in energy. Additionally, readers are referred to recent reviews that describe the optimal TE properties of conjugated polymers and materials, which may be n-type and p-type based on the identity of the dopant [49]. The following Table 1 displays the maximum PFs for doped conjugated polymers, which are typically found empirically [50]. Polymer-based TE materials and their optimization have been subjected to several recent reviews, with particular attention paid to organic TE materials [49], preparative aspects of composites [51], a survey of organic TE materials, and fundamental physics such as carrier motilities and the interdependencies of TE parameters and electronic aspects of the system [52,53].

### 2.2. Thermoelectric Characteristics of Polymer Composites and Blends

Blending with a small molecule, polymer, or nanoparticle addition is one of the simplest and most economical ways to change the properties of polymers. Various small molecules and inorganic salts, which are soluble in polymer matrices, have been extensively researched for electronic applications, such as field-effect transistors and organic solar cells [58,59,60]. In situ reduction of inorganic salts to make (nano)particles in the presence of polymers, solution mixing polymers and nanoparticles, or monomer polymerization in the presence of nanoparticles, are the methods used to create composites. Optical characteristics are obtained by the homogeneous and uniform dispersion of nanoparticles in the polymer matrix [61]. Ligand exchange or covalent modification of nanoparticles can be employed to guarantee that two components are miscible and maximize polymer–particle interactions [62]. For TE applications, the polymer can be either conductive (polyaniline, PANI) or insulating (polystyrene, PS), and the nanoparticulate additive can be inorganic (e.g., Bi_2_Te_3_) or organic (e.g., C60 fullerene). The only requirement is that the conductive domain can connect via the material’s active area [63]. The surface of the particles can promote polymer organization (crystallization) to enhance electrical conductivity. Particles of different sizes and aspect ratios, including spheres, rods, and platelets, can be employed. Depending on the relative ratio of materials, the blend can be either n-type or p-type, where both components have TE qualities and are n-type or p-type. They can operate in concert to improve the Seebeck coefficient when opposing carrier types. The Fermi values of two conductive materials must also coincide with reducing the energy barrier for the charge carrier moving between two phases. Therefore, the Seebeck coefficient can be optimized by choosing the right materials, particle size, shape, and polymer–particle interaction. Some of the standard polymer composites used as p- and n-type materials are listed in Table 2.

## 3. Thermoelectric Generators

Thermoelectric generators convert temperature differences directly into electric energy through the Seebeck effect, functioning without moving parts and enhancing their reliability and maintenance ease [65,66]. They are mainly in applications where waste heat is abundant, such as automotive exhaust systems. They can operate efficiently at temperature differences as low as 3 K, with optimal output achieved at higher differentials [65,67]. In building systems, TEGs can be integrated into facades, walls, and roofs to generate power from temperature differentials with facade systems achieving an output of up to 100 mW/m^2^ [68]. They are also utilized in automobile exhaust systems, where higher temperatures produce much energy, with production reaching about 250 W/m^2^ at 40 K differentials [65]. Additionally, TEGs are effective in small-scale applications, such as powering sensors and wearable devices, although the power density is very low in these contexts [23]. Integrating TEGS with other technologies, like photovoltaic systems and hybrid energy-harvesting methods, enhances their efficacy and application scope, particularly in waste heat recovery from sources like internal combustion engines and fuel cells [69].

Thermoelectric generators are classified into various types based on their design and application, including those used for waste heat recovery, space exploration, and remote power generation [70,71]. The performance of TEGs can be adversely affected by factors like temperature gradients and load resistance, necessitating careful design to minimize the power loss [72,73]. Furthermore, the integration of TEGs into systems often requires complex interface circuitry to optimize energy harvesting, which can introduce additional inefficiency [73]. Overall, while TEGs present promising opportunities for energy conversions, their limitations in efficiency and cost-effectiveness pose a challenge for widespread adoption [74]. Seebeck, Peltier, and Thomson effects are the main coupled heat transfer and electrical transport processes that form the basis of the governing equations of a thermoelectric generator (TEG). These formulas explain how electrical currents affect heat movement and how temperature gradients produce electrical power [75].

A temperature difference across the material is proportional to the generated potential.

V α ΔT

V=SΔT

(1) $$S=\frac{\Delta V}{\Delta T}\;\ldots \ldots \ldots \ldots \ldots$$

where:

V = voltage;

S = Seebeck coefficient (V/K);

ΔT = temperature difference between hot and cold sides.

The current density of the TEG can be expressed by:

(2) $$J=\sigma \cdot \left(E+S∆T\right)\;\ldots \ldots \ldots \ldots \ldots$$

where:

J = current density;

σ = electrical conductivity;

E = electric field;

∆T = temperature gradient.

Heat flux in a thermoelectric material is denoted through conduction, Peltier, and Joule heating terms:

(3) $$q=-k∆T+STJ-\frac{J^{2}}{\sigma }\;\ldots \ldots \ldots \ldots \ldots$$

where:

q = heat flux;

k = thermal conductivity;

STJ = Peltier heat transport;

$\frac{J^{2}}{\sigma }$ = Joule heating.

In a TEG module, energy conservation is stated as:

(4) ∆⋅q+J⋅E=0 ……………

This confirms the balance between heat and electrical energy [76,77].

The Seebeck coefficient S, electrical conductivity σ, and thermal conductivity κ are combined into the dimensionless number ZT = S^2^ σT/κ in the traditional figure of merit ZT, which was developed for steady-state electronic conductors. Although ZT is a helpful compact measure for comparing electronic thermoelectric materials under steady-state charge transport, there are a number of issues with its direct application to ionic and thermogalvanic systems [78]. First, rather than consistent electronic conduction, ionic thermoelectric phenomena frequently depend on mass transfer, capacitive charging, or redox processes. Ion accumulation at interfaces and time-dependent charging processes can dominate the measured thermovoltage in capacitive systems; in thermogalvanic cells, temperature-dependent redox potentials cause open circuit voltage, and reaction kinetics and electrode surface area have a significant impact on device performance. A single steady-state electronic figure of merit does not adequately describe the dominating physics since these mechanisms go against the steady state assumptions that are inherent in the calculation of ZT [79]. Second, the thermovoltage and power output can change over durations determined by ion diffusion, double layer formation, or redox kinetics, making ionic systems intrinsically transitory. Thus, practical performance may be overestimated by a metric that ignores temporal dependency. Similar to this, hydrogels add complexity through swelling, solvent concentration, and mechanical limitations, all of which have an impact on ion mobility and electrode interfacial contact [80]. Hydrogels play a vital role in improving the properties of TEGs through their unique mechanical, thermal, and electrochemical characteristics. For instance, ionic hydrogels composed of polyacrylic acid and polyethylene glycol exhibit impressive mechanical strength, stretchability, and self-healing capability, which are essential for wearable applications, achieving an open circuit voltage of 64 mV and a power density of 4.0 mWm^−2^ under a 2.5 K temperature gradient [81]. Additionally, hydrogel-like gelatin methacrylate combined with polyvinyl alcohol demonstrates commendable thermoelectric properties, achieving notable power densities and open circuit voltages, thereby facilitating efficient energy harvesting from low-grade heat [82]. Furthermore, incorporating superhydrophobic features into a hydrogel enhances its durability and functionality, allowing for effective solar energy utilization while maintaining low thermal conductivity [83]. Another approach, a hydrogel-based printing strategy, yielded a TEG with a power density of 1.278 Wm^−2^ at a 50 K temperature difference, showcasing the potential for high-performance applications [84].

**Figure 1:**
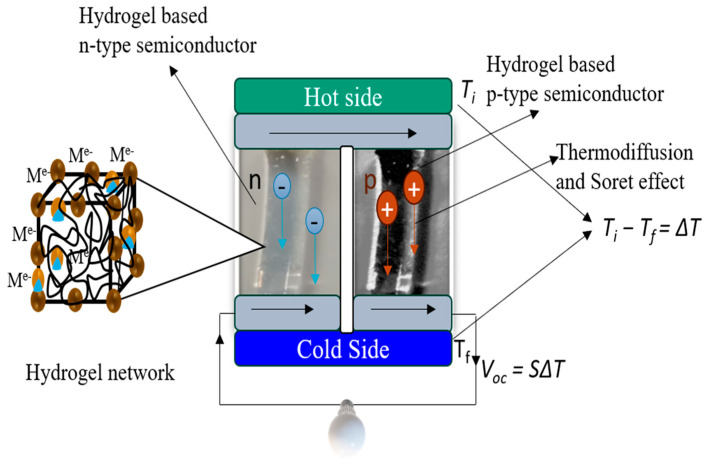
Schematic diagram of hydrogel-based thermoelectric generator cell.

The hydrogel-based thermoelectric generator’s operation is roughly summarized in Figure 1. Mobile ions undergo thermodiffusion (the Soret effect) and redistribute along the temperature axis when a temperature gradient ΔT is introduced across the hydrogel. A net charge separation and an electrostatic potential arise because cations and anions often have differing Soret coefficients and mobilities; in the steady state, this results in an open circuit ionic voltage provided by

$$V_{oc}=S\Delta T$$

where *S* is the ionic Seebeck coefficient [85,86].

## 4. Hydrogels in Thermoelectric Generators

**Figure 2:**
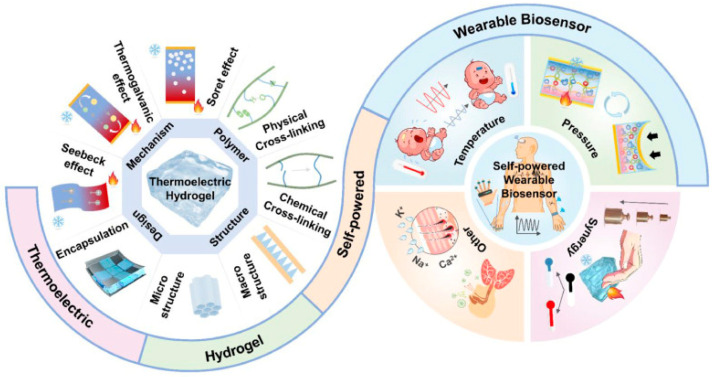
An outline of thermoelectric hydrogels, thermoelectric schematic diagram, structure, design, and application (reused with permission from ref. [108]).

Hydrogels are complex materials with unique physicochemical properties utilized in thermoelectric generators to convert thermal energy to electric energy. They show promise in applications like body monitoring and energy storage, enhancing efficacy and stability in energy-harvesting technology (Figure 2) [87].

Thermoelectric generators face several limitations that hinder their widespread commercialization and efficacy. One significant challenge is the presence of parasitic thermal and electrical resistances, particularly in microelectronic TEGs, which can severely impact performance, especially when using materials with a low thermoelectric figure of merit (ZT) like silicon [88]. While recent advancements have improved efficiencies, TEGs still cannot match the performance of traditional technologies like steam engines, limiting their application scope to niches where other technologies are less effective [89]. Lastly, the economic viability of TEGs is often challenged by the cost per watt compared to other energy sources, particularly in scenarios where fuel costs are low [90]. Hydrogel significantly enhances the performance of thermoelectric generators (TEGs) by addressing limitations such as mechanical flexibility, ionic conductivity, and thermal efficiency. Incorporating hydrogels allows for the fabrication of flexible TEGs that maintain high electrical conductivity and Seebeck coefficients, with minimal degradation in performance due to the hydrogel’s ability to stabilize nanomaterial dispersions [84]. The development of triple-network hydrogels has further improved the ionic conductivity, achieving values up to 168 mS cm^−1^, essential for efficient energy conversion from low-grade heat [91]. Moreover, innovative designs utilizing hydrogels with oppositely charged carriers have demonstrated enhanced thermoelectric performance, showcasing the potential for improved efficiency in wearable devices [92]. Finally, integrating n-type and p-type hydrogel thermoelectric cells has led to significant advancement in voltage output and power generation, highlighting the versatility of hydrogels in thermoelectric applications [93].

Nowadays, hydrogels, especially conductive polymer-based hydrogels, are very popular for their application in wearable electronics and flexible devices because of their biocompatibility, flexibility, and tunable conductivity [94]. Current development indicates their utilization in wearable electrochemical biosensors, which act as biomolecule carriers and support non-enzymatic sensing in integrating nanomaterials [95]. Developing hydrogels, such as polyacrylamide and quaternary ammonium chitosan, have promising characteristics in strain sensing and electromagnetic interference shielding by increasing mechanical durability and antimicrobial properties [96]. When hydrogels are doped with polysaccharides, their toughness and electrical conductivity increases [97]. A conductive, multifunctional hydrogel is suitable for soft robotics and energy storage and has potential for advanced technology [98,99]. Additionally, the discovery of conductive polymer nanoparticles can find application in biocompatible formulations, enhancing the integration of these hydrogels in bioelectronics [100,101].

Several studies highlight the advantages of different hydrogel compositions, such as gelatin methacrylate and polyvinyl alcohol, achieving an open circuit voltage of 64 mV and a power density of 4 mWm^−2^ under a 2.5 k temperature gradient, which can host redox couples and exhibit good thermoelectric properties, including a Seebeck coefficient up to 3.26 mVk^−1^ [82]. Ionic hydrogels, composed of polyethylene glycol and polyacrylic acid, show impressive mechanical resilience and self-healing properties, making them fit for low-grade waste heat harvesting [81]. Innovative hydrogel-based printing strategies have been developed to fabricate flexible TEGs with minimal loss of electrical conductivity, achieving power densities of 1.278 Wm^−2^ under substantial temperature gradients [35]. Furthermore, unique thermophiles using hydrogels with oppositely moving ionic carriers achieve a combined Seebeck coefficient of 6.18 mV/k, enhancing thermoelectric performance significantly. These advancements suggest that hydrogels can effectively convert thermal energy into electrical energy, paving the way for sustainable energy solutions in soft electronics [83].

The scope of improvement in hydrogel-based thermoelectric generators is significant, as evidenced by recent materials composition and structural design advancements. Enhancing the performance and scalability of hydrogel-based thermoelectric generators involves several key advancements. Material optimization improves efficiency by strengthening the hydrogel matrix’s electrical conductivity and Seebeck coefficients [84]. Refining hydrogel-based printing techniques can lead to a more precise and efficient fabrication process, ensuring uniformity and stability in the printed layers, which is vital for maintaining performance. Addressing thermal management by improving thermal stability and heat dissipation properties of hydrogel-based TEGs can prevent degradation over time and enhance their efficacy. Finally, achieving scalability through cost-effective and optimized manufacturing processes is important for the widespread adoption of these devices. By reducing the production cost while maintaining superior performance, hydrogel-based TEGs can be effectively integrated into diverse applications, accelerating their practical implementation [84]. Several strategies can be employed to improve the conductivity and reduce the thermal conductivity of gels, particularly silica aerogels. Firstly, nanoparticles such as graphene, carbon nanotubes, and metal oxide or metal nanoparticles are incorporated as fillers, which are efficient conductive pathways within the gel. These nanomaterials bridge gaps in the hydrogel network, significantly boosting its electrical performance [102,103]. Optimizing the polymer matrix by utilizing polymers with conjugated structures, like polyaniline or polypyrrole, can enhance the gel’s intrinsic conductivity due to their ability to facilitate electron transport [103]. For ionic gels, increasing ionic conductivity is another important strategy. This can be achieved by incorporating ionic liquid or salt, which introduces more charge carriers and improves the gel’s overall charge transport capability. Together, these strategies enable the development of highly conductive hydrogels tailored for advanced energy applications [104]. On the contrary, reducing thermal conductivity in hydrogels can be achieved through several approaches. Introducing porosity by creating a porous structure within the gel is an effective method, as it traps air, which is a poor conductor of heat [105]. Another strategy is using low-thermal-conductivity fillers, such as silica aerogel or cellulose nanofibers, which help further reduce the gel’s thermal conductivity [105,106]. Additionally, adjusting the crosslinking density of the polymer network can significantly influence the thermal conductivity. Specifically, a lower crosslinking density tends to result in reduced thermal conductivity, making it a critical parameter for optimizing the thermal properties of hydrogels [107].

## 5. Classification and Fundamental Limits of Hydrogel-Based Thermoelectric Systems

**Figure 3:**
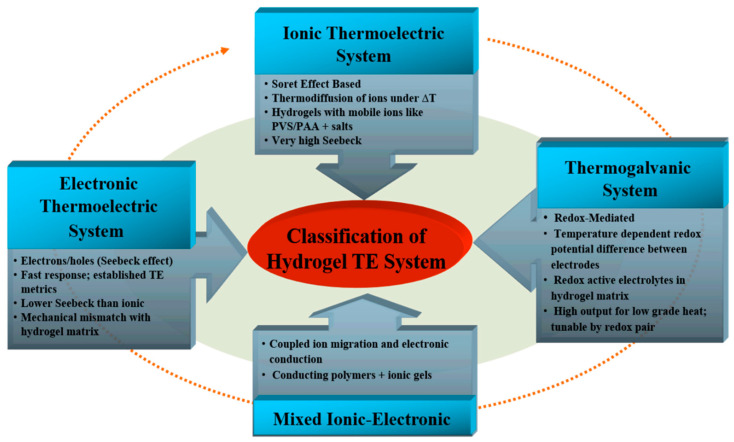
Classification and properties of hydrogel-based thermoelectric (TE) system.

The major charge carriers, operating mechanisms, and device design of each of the four practical classes of hydrogel thermoelectric devices are shown in Figure 3.

(a) **Ionic (thermo-diffusive)**—Differential ion migration is driven by temperature gradients, resulting in an open circuit voltage whose magnitude and sign are controlled by the polymer network and ion–polymer interactions.

(b) **Thermogalvanic (redox)**—Heat is converted to electricity by temperature-dependent redox potentials at electrodes; redox coupling, electrode kinetics, and electrolyte confinement all affect performance.

(c) **Electronic (conducting-polymer/inorganic composite) and (d) mixed ionic–electronic/protonic**—Electronic conduction embedded in hydrated networks is provided by distributed inorganic TE particles or conductive polymers (PEDOT: PSS, polyaniline); design strikes a compromise between mechanical compliance, conductivity, and Seebeck [36,45].

Table 3 presents the hydrogel-based thermoelectric materials along with their roles and performance. Hydrogel (ion-based) systems are fundamentally distinct from traditional inorganic TEs in several aspects. Instead of using electrons or holes, hydrogel systems rely on ions, which are massive, solvated species. This results in very large Seebeck coefficients (mV K^−1^) because gradients in ion concentration can generate large open circuit voltages, but ionic conductivities are orders of magnitude lower than electronic conductivities, which limits the power delivered. While inorganic electronic TEs function in a steady state with rapid electronic transport, ion diffusion is sluggish and frequently non-steady during continuous operation, resulting in time-dependent responses and saturation at greater ΔT [109,110]. Ionic diffusion of hydrogel TE materials has several intrinsic restrictions compared to electron transport. Ionic conduction also strongly relates to the solvent and polymer matrix, resulting in performance that is reliant on temperature and humidity. Ionic mobilities are lower, limiting current and power density. Hydrogels can lower efficiency and lower ΔT overactive regions due to their high heat capacity and frequently greater thermal conductivity paths (water channels). Although redox (thermogalvanic) systems can produce more power, they have limited cycle life in soft matrices, electrode corrosion, and redox shuttle losses [111,112].

## 6. Properties of Hydrogel-Based Thermoelectric Generators

TEGs cannot meet the requirements for wearable applications and bionic systems because of their packaging barriers and toxicity. When integrated with TEGs, hydrogels provide unique flexibility and bioactivity, enabling it for wearable applications. This section will illustrate the advantages and application of hydrogels compared to other materials in this research domain.

### 6.1. Flexibility

This flexibility is achieved through specific structural designs and material compositions. The creation of polymer chain entanglements is facilitated by the low-temperature gradient-directed freezing process, which adds to the hydrogel’s high mechanical strength and ductility. In addition to facilitating ion transport, the directed freezing technique produces an ordered pore structure with parallel ion-channel alignment, which enhances the mechanical characteristics of the composite hydrogels [144].

Devices and TE materials can convert heat from different heat sources, but traditional TE materials, mainly inorganic materials, suffer due to their rigidity and weight. This limits their applications when they come into contact with bent and flexible devices such as the human body [145]. Flexible TE devices and materials can efficiently interface heat sources to optimize heat use. Nowadays, conductive polymers, metal nanowires, and organic–inorganic hybrids are used to make flexible thermoelectric materials [146]. Specific devices use flexible substrates such as polyimide (PI), polyvinylidene difluoride (PVDF), or polydimethylsiloxane (PDMS), or flexible architecture (like hinge-type designs) that can also bend [147]. However, the material’s tensile strain (usually less than 20%) severely limits the stretchability of these flexible TE materials [148]. Additionally, the hydrogel network’s water can be a lubricant, lowering the force required for deformation. The stretchability (strain > 200%) of hydrogel-based thermoelectric materials and devices is noticeably better than that of other thermoelectric materials and devices [149]. A summary of the literature review on FTEGs is presented in Table 4.

### 6.2. Adhesion

Existing TE materials have shown enhanced usage of heat from irregular sources as alternatives to conventional rigid TEDs [155]. Nevertheless, flexible TEDs invariably experience poor contact phenomena such as air gaps when they interface with extremely uneven heat source surfaces, particularly in portable applications. Heat transmission efficiencies are limited because of these spaces between the heat source and the TED, which prevent optimal physical contact from forming [156]. Hydrogels are now the perfect candidate materials for creating flexible, wearable devices because of their stretchability, sensitivity, and adhesiveness. Additionally, it is simple to attach and detach flexible devices based on adhesive hydrogels as needed [157].

**Figure 4:**
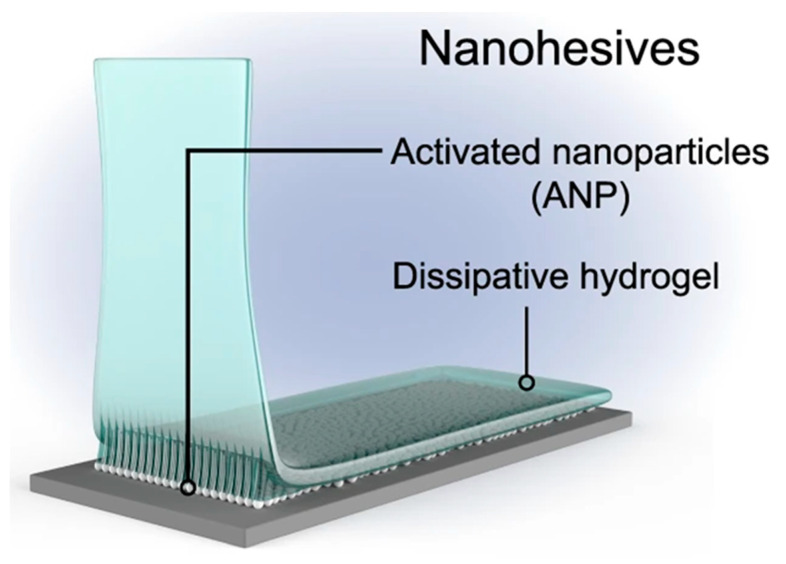
A layer of activated nanoparticles (ANP) and a dissipative hydrogel make up the nanohesives [158].

In order to provide conformal electrical/ionic contact and stable mechanical coupling for wearable thermoelectric generators (skin contact applications), hydrogels adhere to thermoelectric generators (TEGs) by forming intimate, frequently reversible interfacial bonds through catechol (mussel-inspired) chemistry, hydrogen bonding/electrostatic interactions, dehydration-driven contact, mechanical interlocking, and nanoparticle-mediated “nanohesive” bridging shown in Figure 4 [158]. Strong bidentate coordination, covalent bonds (after oxidation), and reversible hydrogen bonds are formed by catechol groups on polymer chains with metal oxides, metals, and organic surfaces; this results in strong wet adhesion for hydrogel electrodes and interfaces, enhances electrode contact and lowers interfacial resistance when used in hydrogel TEGs. Through many hydrogen bonds and ionic pairing, polar polymer networks (such as polyacrylamide and polyvinyl alcohol) interact with surface hydroxyls, amines, or charged electrodes to form numerous weak connections that add up to significant macroscopic adhesion [159].

### 6.3. Self-Healing Ability

**Figure 5:**
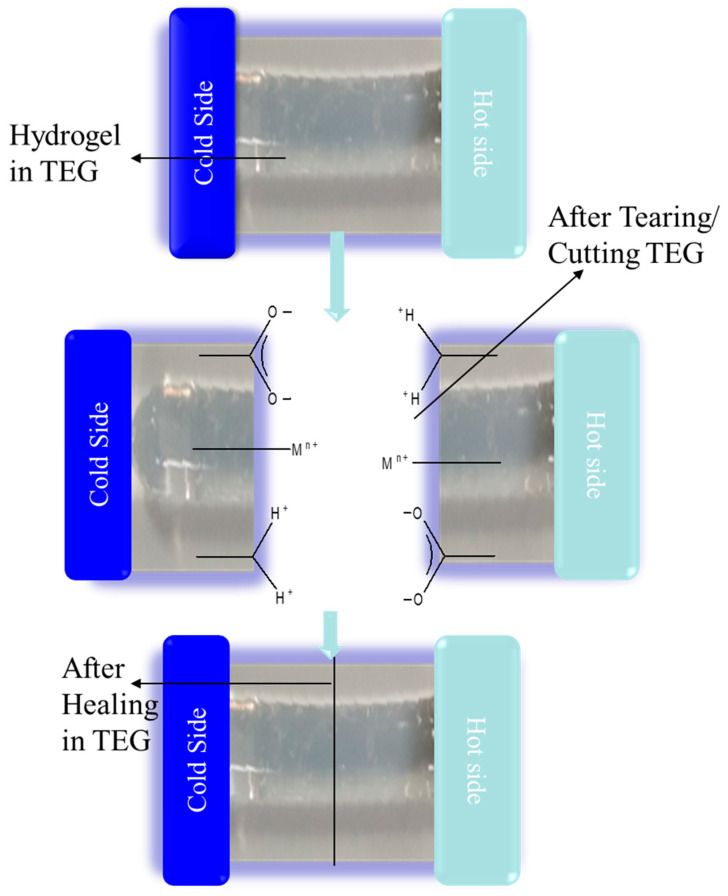
Self-healing of hydrogel in TEG.

The performance of thermoelectric generators can decline due to fatigue, damage, or corrosion while they are used. If this device could self-heal like a biological process and retain its mechanical performance like its actual characteristics, it could be operated long-term [160,161]. The capacity of the hydrogel to independently regain its mechanical structure and electrical/ionic conductivity following injury (cut, fractured, stretched, or pierced) is referred to as self-healing. Because thermoelectric devices frequently experience mechanical stress, heat cycling, and repetitive deformation in wearable or flexible electronics, this feature is particularly significant. Because their polymer networks are composed of reversible, dynamic bonds (hydrogen bonds, ionic interactions, host–guest, metal–ligand, or reversible covalent bonds) that re-form after mechanical damage, hydrogel-based thermoelectric generators (i TEGs) exhibit self-healing. This restores the continuous ionic pathways necessary for ionic Seebeck (thermo-diffusive) voltage generation. According to recent research, self-healing hydrogels may restore both mechanical integrity and ionic conductivity/Seebeck coefficient, allowing for the development of long-lasting wearable IT devices [162,163]. Self-healing could be operated by a hydrogel using two mechanisms, depending on the self-healing processes. The dynamic covalent reaction is one in which external stimuli are required to maintain the process. Non-covalent interaction is another process that permits autonomous self-healing [164,165]. Reversible hydrogen bonding interactions and chain entanglement are the main mechanisms behind the self-healing of these ionic hydrogels. According to Mi Fu et al., polyacrylic acid (PAA) and polyethylene glycol (PEO) doped with sodium chloride form a physically crosslinked network that makes up the PAA-PEO-NaCl ionic hydrogels. The hydrogels can regain their initial mechanical and thermoelectric characteristics due to chain entanglement and reversible hydrogen bonding between carboxylic acid and ether groups. By enhancing polymer chain mobility and encouraging hydrogen bond re-bonding, higher humidity might hasten the healing process [81]. A dynamic physical crosslinking network is present in the hydrogels. The material can return to its initial condition shown in Figure 5 when an external force is removed because the hydrogen bonds can re-form. This dynamic quality makes it easier to repair damaged characteristics [166].

## 7. Working Mechanism of Hydrogel-Based TEGs

In thermoelectric generators (TEGs), hydrogels use phase transitions, ionic conduction, and thermodiffusion to transform low-grade heat into electrical power. They are versatile, effective, and appropriate for wearable energy harvesting because of their special water-rich polymer networks, which allow ion mobility, entropy-driven charge separation, and redox reactions at electrodes. Hydrogels have a high water content, which improves flexibility and mechanical adaptability, providing a medium for ion transport and for ionic conductivity to be tailored by modifying polymer composition and additives [167]. When heated, certain hydrogels experience volume phase transitions (VPTs). A significant ion entropy difference is produced as a result of this transition, which modifies ion distribution. The output voltage is increased by the entropy difference, which also improves thermodiffusion and redox reaction efficiency. Ionic thermodiffusion—the movement of ions under a temperature gradient—is made possible by hydrogels. The Seebeck coefficient (Se), a crucial metric for evaluating thermoelectric performance, is largely influenced by this phenomenon.

**Figure 6:**
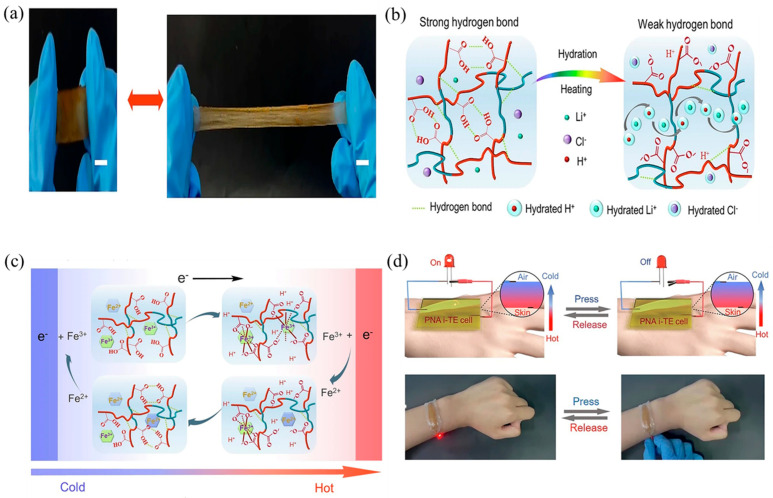
(**a**) Images of a reversible, stretchable PNA i-TE cell; (**b**) schematic representation of the interaction processes in PNA hydrogels’ as-fabricated i-TE materials during the VPT; (**c**) schematic representation of the huge thermopower PNA i-TE cell’s ion coordinate processes; and (**d**) schematic representation of the PNA i-TE cell using body heat to illuminate an LED.

According to Xiaofang et al., p(N-acryloylsemicarbazide-co-acrylic acid) (PNA), a temperature-responsive supramolecular hydrogel depicted in Figure 6a, may be utilized as an ionic thermoelectric cell that combines favorable mechanical and electrochemical characteristics. With a temperature differential of 50 K, a single PNA i-TE cell may produce 2.04 volts of thermopower. The PNA i-TE cell’s Seebeck coefficient (Se), figure of merit (ZT), and specific output power density (Pmax = ðΔT^2^2) may all reach up to 40.9 mV K^−1^, 35.2 mW m^−2^ K^−2^, and 1.33, respectively. This hydrogel makes use of supramolecular hydrogen bond networks (Figure 6b), which provide an adequate supply of ions inside the hydrogel matrix and offer swelling resistance. Robust mechanical qualities are also influenced by these networks. By giving ions favorable routes, these networks promote ion transport, resulting in high-efficiency ionic thermodiffusion over a temperature gradient. The hydrogels’ capacity to retain water is improved by the addition of lithium chloride (LiCl), which is essential for ionic conductivity. The hydrogels seen in Figure 6c have high mechanical characteristics because the Fe^3+/2+^ redox pair functions as ionic crosslinkers in addition to contributing to the thermogalvanic action. In contrast to the PNA hydrogel without ions, the coordination of Fe^3+/2+^ ions causes the hydrogel network to self-strengthen, which can restrict the ion channels and reduce ionic conductivity. The significant ion entropy difference produced by this VPT process increases the velocity of ionic thermodiffusion as well as the efficiency of the redox reaction. Heat causes the carboxyl groups of PAA chains to separate from dimers and the inter/intramolecular hydrogen bonding decreases, making the polymer chains more hydrophilic. By facilitating the protonation of carboxyl groups, the VPT offers metal ion binding sites. Because the carboxyl groups connect more readily with Fe^3+^ than with Fe^2+^, thermopower is increased and the entropy difference in the Fe^3+^ redox pair is increased. A single PNA i-TE wristband can power an LED at an ambient temperature of about −5 °C by harvesting human thermal energy without the need for a voltage amplifier, as shown in Figure 6d [168].

Hydrogel-based TEGs depend on ion transport, in contrast to conventional semiconductor TEGs. Applying a temperature gradient causes ions (such as Cu^2+^, a Seebeck potential is produced when Cl^−^) diffuse at various rates. Compared to inorganic materials, this ionic Seebeck coefficient can reach values as high as 40.9 mV/K. Metal-ion redox couples, such as Cu/Cu^2+^, are frequently integrated into hydrogel TEGs. Redox processes at electrodes are facilitated by temperature-driven ion migration, which maintains constant current output. Using the MXene/PVA (polyvinyl alcohol) hydrogel, Dezhuang Ji et al. present a continuous-output ionic thermoelectric (i-TE) system in which a three-serial-connected module can provide an output voltage of 26 mV with a temperature differential of 6 °C [169].

**Figure 7:**
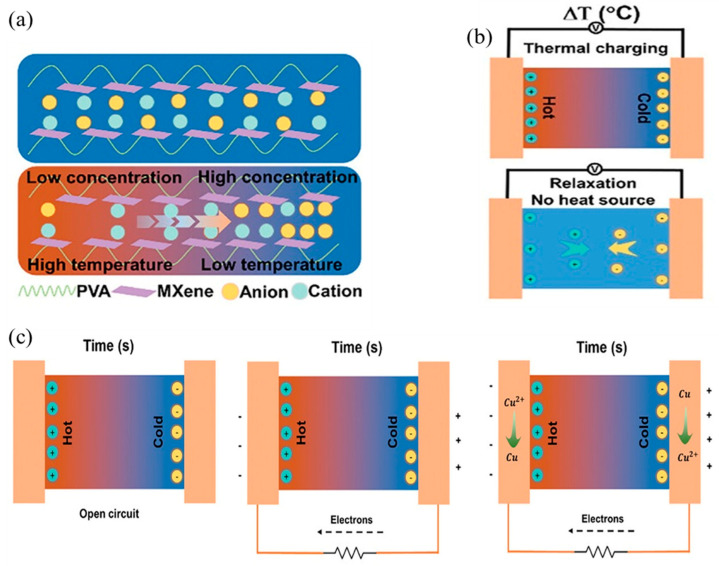
(**a**) An example of how the ionic thermoelectric effect works; (**b**) diagrammatic representation of thermal relaxation and charging, the change in voltage over time during samples’ cyclic thermal charge and relaxation; and (**c**) an example of a continuous current output in principle.

The mechanism of the ionic thermoelectric effect in the MXene/PVA hydrogel system is schematically shown in Figure 7a. The Soret effect is the fundamental functional mechanism of the i-TE effect in ionic systems. Thermophoresis causes ions to move from the hot side of the hydrogel to the cold side when a temperature gradient is placed across it (Figure 7b). Ion concentration on the cold side rises as a result of this action. An induced electric field and a concentration gradient are produced by the buildup of ions on the cold side. Ions then return from the cold side to the hot side as a result of these forces. Eventually, an equilibrium condition is attained when the forces resulting from the concentration gradient and electric fields are balanced by the thermophoresis force. Cu^2+^ and Cl^−^ ions serve as the main entropy carriers in the CuCl_2_ salt-infused MXene/PVA hydrogel system. Cl^−^ has a greater absolute thermopower and a negative net thermopower (N-type i-TE system) because it has a lower hydration energy and is more thermally sensitive than Cu^2+^. The hydrogel’s MXene content is crucial for adjusting the ion diffusivity through interactions. Through electrostatic interactions, MXene surfaces, which have a zeta potential of ≈−35.4 mV and contain negative charges, affect ion mobility. This increases the thermopower by significantly lowering their concentration-induced diffusivity. The MXene/PVA hydrogel is integrated with copper electrodes to provide a continuous current output. Electrons are forced from the cold side to the hot side when an external circuit is connected. As seen in Figure 7c, Cu on the cold side is oxidized to generate Cu^2+^, whereas electrons on the hot side reduce Cu^2+^ to Cu. The Cu/Cu^2+^ redox reaction is completely reversible. The concentration gradient causes the freshly produced Cu^2+^ to migrate to the hot side, creating a full loop in which ions move within the internal MXene/PVA hydrogel and electrons flow via the exterior circuit. Unlike capacitive i-TE devices, which gradually decrease to zero voltage, this technique guarantees ongoing functioning [169].

Cu/Cu^2+^ couples are appealing because of their high redox potential and quick kinetics; however, electrode surface changes make them unstable after thousands of heating–cooling cycles. Because of surface oxidation and ion depletion, repeated cycling may cause a progressive loss of reversibility. The development of passivation layers (such as CuO and Cu_2_O) and corrosion at the electrode–hydrogel interface are major obstacles for Cu-based thermogalvanic cells. Long-term performance is hampered by these processes, which decrease active sites for electron transport. According to recent research, bilayer passivation techniques such as anodic coordination polymerisation can greatly increase copper’s oxidation resistance and prolong its useful life [170]. Chelating ligands or stabilizers can be employed to preserve Cu^2+^ solubility and avoid precipitation, hydrogel composition can be engineered, and protective coatings or polymer layers can be added to reduce corrosion [171].

## 8. Application of Hydrogel-Based Thermoelectric Generators

Hydrogel-based thermoelectric devices are appropriate for wearable applications because they can collect low-grade heat energy from the surroundings [172]. They can efficiently capture steady heat from the human body and transform it into electrical power for low-tech medical equipment, like heart rate and blood pressure monitors [173]. In order to survive more than 10,000 mechanical cycles and stay hydrated for days or weeks, wearable health monitors usually need 10–100 μW of continuous power and a small 2–5 K skin–air gradient. Onboard energy buffering and charging necessitates ionic/electrochemical stability over hundreds of charge cycles and aggregated outputs on the order of 0.1–1 mW, which may be accomplished by module stacking under 5–10 K gradients. These ranges may provide realistic aims for the development of hydrogel ionic TEs and are in line with recent experimental and review research [45,174,175]. This section will discuss some of the applications of hydrogel-based thermoelectric devices.

### 8.1. Human Health Monitoring

**Figure 8:**
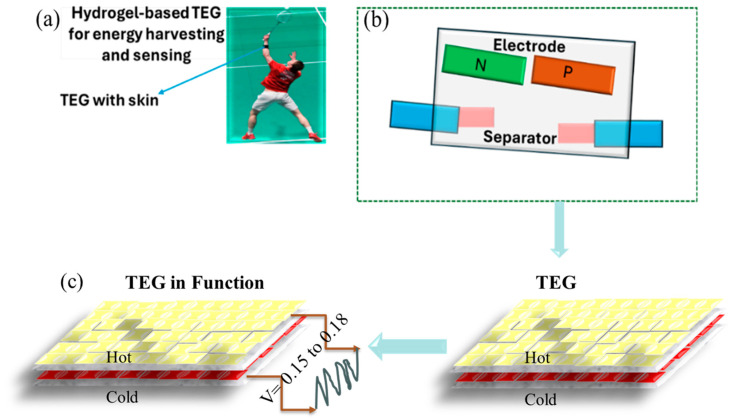
Hydrogel-based thermoelectric generator for wearable applications. (**a**) Schematic diagram of how hydrogel-based thermoelectric devices work for wearable applications. (**b**) Description of the integration of stretchable thermocells. (**c**) The voltage was recorded as it varied over time, affixed to the wrist while under strain.

Different impulses from different body regions can be tracked using thermoelectric skin sensors or skin adhesive components [176]. The primary benefits of employing hydrogels for human monitoring applications are their high sensitivity to stretch durability under prolonged cyclic mechanical loads and their usefulness because of their high strength (Figure 8a) [137]. Figure 8b shows the very flexible quasi-solid state ion hydrogels used to make wearable self-powered sensors based on TEDs, which have a fracture strain of 1000% and demonstrate good thermoelectric sensitivity to bending and strain after several cycles. Furthermore, using flexible electrodes and membranes, self-powered TEDs are combined with 14 pairs of hydrogel-based p-n legs. When self-powered sensors are affixed to a human wrist joint at 4.1 °C, the V fluctuates marginally between 0° and 90°, producing V changes demonstrated in Figure 8c [135].

### 8.2. Storage of Energy

Wearable self-charging gadgets combine energy storage units, power management tools, and energy harvesters into a single platform. They can store electrical energy for later use while harvesting energy from the environment or the human body. However, the processes separating energy storage and transmission make it challenging to create integrated dual-function devices [121]. Hydrogel-based thermoelectric combined devices that can combine thermoelectric and supercapacitor functions in one device have generated much interest lately [142].

**Figure 9:**
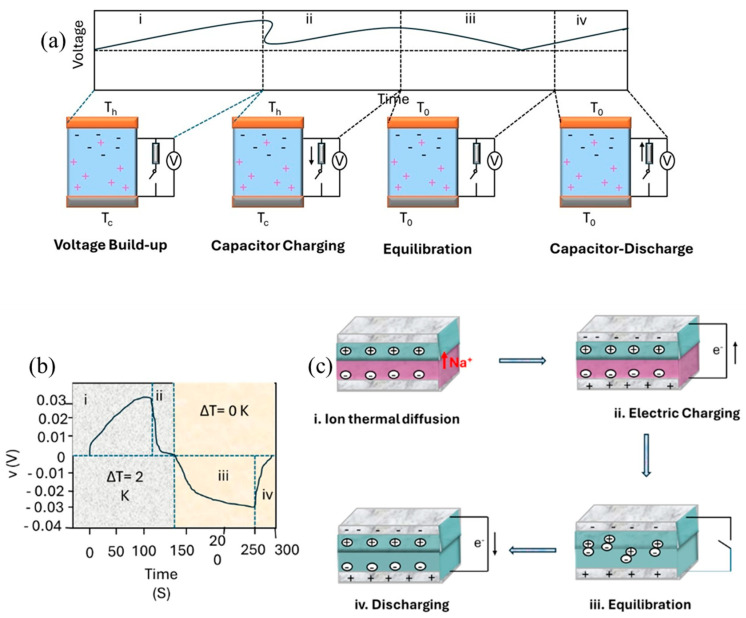
Hydrogel-based thermoelectric generators for energy storage and harvesting. (**a**) Diagram and mechanism for the i-TE capacitor. (**b**) The voltage of the ionic thermoelectric supercapacitor was measured at the time of charging and discharging. (**c**) Diagram for transport carrier mechanism [121].

One benefit of employing ions as energy carriers in ionic thermoelectric cells is their ability to achieve a high voltage of 1–5 V at room temperature. Figure 9a shows four steps to a capacitor mode: voltage buildup, charging, equilibration, and discharge [140]. The all-hydrogel ion thermoelectric supercapacitor allows heat to be converted into electricity while it is stored in the flexible hydrogel electrodes. Four steps may be distinguished in the charging and discharging process of the ion thermoelectric supercapacitor: (i) cations migrate directionally within the hydrogel when T is applied; (ii) cation-induced electrons accumulate near the positive electrode and are transferred through the external circuit; (iii) electrons and holes are stored in the two electrodes when the external circuit and T are removed; (iv) electrons and holes neutralize one another when the external circuit is reconnected [121]. This is shown in Figure 9b,c, respectively.

### 8.3. Interaction Between Machine and Human

**Figure 10:**
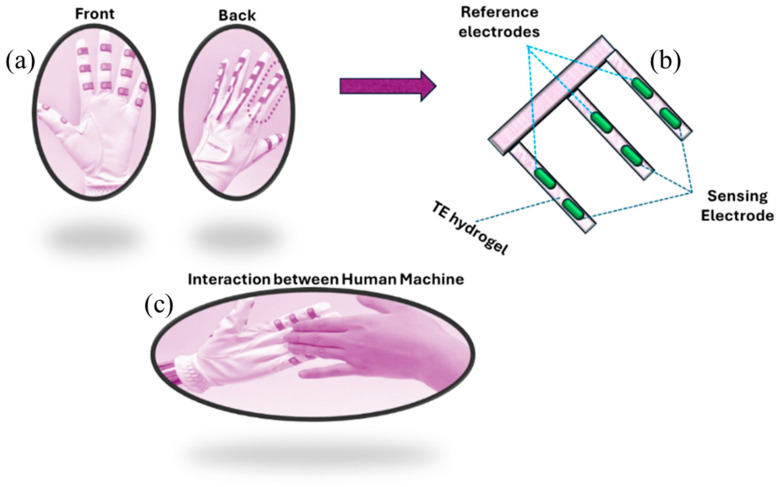
Interaction between human and machine depends on hydrogel-based thermoelectrics. (**a**) Bright gloves incorporating thermal sensors; (**b**) each finger contains a flexible thermal sensor; and (**c**) interaction with a brilliant glove featuring advanced ionic hydrogel thermal sensor arrays designed to enhance the human–machine interface seamlessly.

Human–computer interaction has grown in importance in recent years due to technological advancements that allow robots to communicate with humans more organically and intuitively [177,178]. Because of their intrinsic stiffness, a conventional semiconductor-based device for human–computer communication remains outdated. The mechanical mismatch between silicon-based bioelectronics and human tissue makes seamless, long-term communication and interaction difficult for these materials and devices, even though advancements in materials engineering and device design have made them sufficiently thin to achieve flexibility [179,180]. The next generation of human–computer interfaces will communicate seamlessly thanks to the hydrogel’s exceptional compatibility and tissue-like properties. Compared to other thermoelectric materials, hydrogel-based thermoelectric materials are more sensitive and valuable due to their high S (tens of mVK^−1^). For example, a flexible thermal sensor array with a sensitivity of 2.7 mVK^−1^ for spatial temperature detection was created using high-performance ion thermoelectric hydrogels with an S of 24.17 mVK^−1^. An innovative glove with heat sensing as a human–computer interface has been developed (Figure 10a–c) due to the thermally driven ion transport within the hydrogels, which may be a promising option for thermal sensing [113].

## 9. Challenges for Hydrogen-Based TEGs

Hydrogel-based thermoelectric generators (TEGs) are an emerging technology with significant energy-harvesting and conversion potential. However, several challenges must be addressed to enhance their performance and applicability. Over the last decades, initiatives to enhance electric output in low-grade heat ranges have yielded promising results, mainly using hydrogels and related materials. Attention is shifting toward achieving real device power, which remains comparatively low against other compact energy conversion devices. Discrepancies in experimental results and calculation methods pose challenges for future advancement. Regarding the experiments, the temperature gradients exhibited considerable variation in magnitude and orientation (in-plane and out-of-plane) across different scenarios. Some reports indicated very high thermopower outputs achieved with minimal temperature gradients (less than or equal to 1 K) or measured under in-plane arrangements [117,172,181].

In order to highlight fundamental material constraints, much of the hydrogel-based thermoelectric research describes performance under large ΔT (30–50 K). However, practical wearable harvesting must function with skin–air gradients of ~2–5 K, which significantly decreases useable power. The focus of recent work has switched from reporting just big ΔT benchmarks to lowering thermal conductivity, optimizing thermopower per unit ΔT, and designing device topologies that magnify minor gradients in order to solve this. Device strategies include thermal concentrators, active/passive cooling on the cold side, and hybrid integration with solid TE legs to increase voltage; structural strategies include porous networks and low k scaffolds to increase thermal resistance throughout the device; and material strategies include ion selection and regulation to maximize ionic Seebeck coefficients and phase transition or crystallinity control to stabilize ion diffusion. Future wearable designs should concentrate on these methods as they have shown encouraging results of increased output under practical ΔT [168,182].

The inaccuracy in thermopower characterization becomes evident when lowering the temperature difference, as systematic errors may overshadow the results. A practical approach is to evaluate the thermopower across small and moderate temperature differences (1 to 50 °C). Performing in-plane measurements in an open environment (lacking strict encapsulation) can lead to incorrect interpretations of electric responses, as solvent effects may become more significant than solutes, potentially causing hydro-voltaic phenomena. Therefore, ensuring reliable encapsulation is important for precise thermopower measurement and preventing water loss, thereby upholding long-term capability. Vario’s encapsulation techniques, including soft substrate sealing [183,184], vacuum heat sealing [185,186], and hydrophobic elastomer coating [187], have been implemented for hydrogel-based i-TEs to date. Moreover, the choice of electrode materials (like inert electrodes, gold and carbon, and redox-active electrodes such as copper and PANI) primarily determines the operating mechanism and necessitates careful selection and study [186].

Intrinsic thermoelectric behaviors resulting from specific redox reactions or the gradual corrosion of electrode materials must be considered when assessing the total thermopower of the device, as the latter can negatively impact long-term stability and reversibility. It is essential to clearly outline the measurement of the conditions, and accurate T values with multi-temperature gradients are critical for evaluating thermopower. Due to the different technical foundations of TGCs and TDCs and their deviation from the solid TEs, a reliable and predictable model is necessary to enhance the accuracy in assessing a new figure of merit, along with the power density and energy conversion efficiencies. Various methods have been used to calculate the i-TEs’ performance, making it difficult to draw fair comparisons between challenging studies. Lastly, the lack of a comprehensive theoretical framework limits understanding of hydrogel-based i-TEs and further development of i-TE design [167]. As the concentration of ions increases and soft, charged, and confined interfaces (like hydrogel polymers) are incorporated into i-TE systems, predicting Debye length, ion solvation, and the local dielectric constant becomes challenging with traditional models. Significant gaps exist in understanding the micro-level interactions between water, hydrogel polymers, and ionic species, particularly with nonequilibrium thermal fields and water–polymer binary systems. This system’s understanding of thermo-electrochemical phenomena is ambiguous, especially without in situ quantitative data. Especially regarding TDCs, while it is widely accepted that the different mobilities of anions and cations and the resulting asymmetric charge distribution resulting from a temperature gradient contribute to thermopower, there is a lack of experimental evidence demonstrating the difference in ion mobilities [185]. At the same time, other findings suggested that this rationale was inadequate. To sum up, thoroughly examining the hydrogel-based i-TEs’ mechanism is essential for future research.

## 10. Future Prospect and Conclusions

This review emphasized recent research developments in this area, concentrating on the distinctive characteristics of hydrogels, such as their high flexibility and biocompatibility, that render them appropriate for wearable thermoelectric devices and robotics. Although there have been improvements in materials, device performance, and durability since the concept first emerged in 2016, a notable gap still exists between research outcomes and real-world applications. Thermoelectric devices that utilize hydrogels can fulfill the requirements for self-powered sensing and human–computer interaction; however, there are some challenges in the field of engineering and device design.

To provide consistent performance in a variety of climates, environmentally tolerant conductive hydrogels (ETCHs) with anti-freezing, anti-drying, and anti-swelling qualities can be developed and used for practical TEGs. Hydrogels that are self-healing, weldable, and double-network-encapsulated may exhibit improved thermoelectric performance and better water retention. Machine learning and artificial intelligence are expediting the identification of high-performance thermoelectric materials by predicting Seebeck values, optimizing compositions, and directing experimental synthesis. Device designs may be optimized using sophisticated multiphysics modeling that includes mechanical deformation, ion transport, and heat transfer. Deep learning in conjunction with multiphysics simulations confirmed by experiments may offer predictive insights into thermoelectric generator performance.

For several reasons, particularly in energy conversion, flexible electronics, and medicinal applications, the ongoing research into hydrogel-based thermoelectric materials with high σ and S is important. Enhancing S and σ significantly improves the device’s energy-collecting and conversion capabilities and overall thermoelectric performance, including S^2^σ. While σ is relatively low, achieving a high S is within reach, and vice versa. To achieve high S^2^σ, innovative techniques are being developed, merging S_i_ and σ [139]. Powering various electronic devices while meeting practical application requirements remains challenging. Further research is needed to achieve a balance and improve S^2^σ. Hydrogel-based thermoelectric materials with high S (tens of mV K^−1^) have been extensively used in wearable electronic products, surpassing other thermoelectric materials and devices. However, the challenge of achieving output densities suitable for real-world applications remains. To overcome this, it is important to explore the potential of integrating various bioenergy harvesting and flexible energy storage systems technologies, such as TENGs, PENGs, hydropower, BF, etc., to create self-charging power sources. This will increase the output density of these systems and open up an excellent range of applications. For self-powered wearables or other applications, designing hydrogel-based TEDs usually entails preserving their effectiveness under repetitive mechanical deformation. As the electrolyte and/or electrode in these devices, the hydrogel is more extensible than other components, frequently resulting in interface mismatch or active layer breakage. Hydrogel-based TEDs in conjunction with various electronic devices or hydrogel-based heat sinks in conjunction with commercial TEDs, for example, are significantly stiffer than hydrogels and do not experience significant strain when subjected to mechanical deformation. It is of utmost importance to thoroughly consider the mismatch between the hydrogel and the various kinds of layer, as this can significantly impact the device’s performance. Flexibility can be increased by using island-bridge structures [188] or by making the hydrogel film thinner [189]. Although several strategies for wireless self-powered applications have been proposed, future research should concentrate on creating more adaptable and flexible data transmission solutions that work with thermoelectric systems based on the hydrogel [87].

With unique benefits, including mechanical flexibility, biocompatibility, and superior adhesion, hydrogels for thermoelectric materials and devices are valuable technologies that may be applied directly to the body without needing extra power sources or sensors. Many devices are still experimental and may require additional amplification circuits, even though current research shows that this technology can supply sufficient energy for various devices used in human monitoring and human–machine interaction. However, the potential of this technology can only be fully realized with further research. Improvements in materials engineering, device design, and intelligent system integration are necessary to produce real-world applications. This includes improving thermoelectric performance and stability, investigating novel nanomaterial additions, and optimizing hydrogel composition and composites. Notably, future research should improve the thermoelectric performance and stability of hydrogels and their composites, investigate new nanomaterial additives, and optimize their composition.

Equally important is the need to reduce the size and weight of TEDs while maintaining or improving their performance. This will significantly enhance their portability and adaptability for wearable or implantable applications. Furthermore, the development of wireless communication protocols and systems that are compatible with hydrogel-based devices is essential. Ensuring robust and reliable data transmission in complex and dynamic contexts, such as the human body, is a key aspect of this development.

## Figures and Tables

**Table 1:** The highest power factors for doped conjugated polymers.

Materials	Structure	Dopant	Ỽ [Scm^−1^]	α [µ vk^−1^]	PF [Wm^−1^k^−2^]	Reference
PA	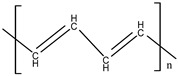	I_2_	44,250	14	2.7 × 10^−4^	[54]
PANI	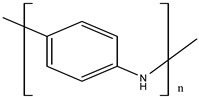	CSA^−^	160	5	4 × 10^−7^	[55]
PPy	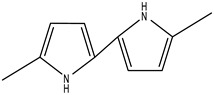	PF_6_^−^	340	10.5	2 × 10^−6^	[56]
Polycarbazole derivatives	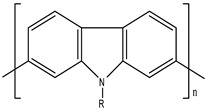	FeCl_3_	160	34	1.9 × 10^−5^	[57]
PEDOT: PSS	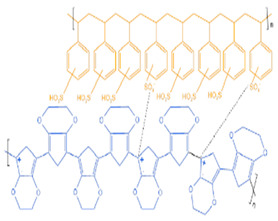	DMSO/EG	890	74	4.7 × 10^−4^	[46]

**Table 2:** Semiconductor materials and their carrier types are frequently utilized in TE applications [64].

P-Type	N-Type
Doped PEDOT	PEDOT
Bi_2_Te_3_	Bi_2_Te_3_
MoS_2_ (bulk)	MoS_2_ single sheet
Polypyrrole	Perylene
Graphene	Graphene
Graphene Oxide	Te
Polythiophene	C60
Polyaniline	SWCNT, MWCNT

**Table 3:** Hydrogel-based thermoelectric materials with their roles and thermoelectric performance.

Materials	Roles	Thermal Conductivity (*κ*) [W m^−1^ K^−1^]	Thermopower (S) [mV K^−1^]	Electrical Conductivity (*σ*) [S m^−1^]	Power Factor (S^2^*σ*) [mW m^−1^ K^−2^]	Reference
	**Ionic (thermo-diffusive)**					
**PQ-10/NaOH**	Electrolyte for ion transport	/	24.17	0.3	/	[113]
**PDDA/AAM/AMPSA/CaCl_2_**	Electrolyte for ion transport	/	3.35	1.16	/	[114]
**PVA/NaOH**	Electrolyte for ion transport	0.42	−37.61	0.00736	/	[86]
**Cellulose/BzMe_3_NOH**	Electrolyte for ion transport	0.18	2.61	3.8	0.42	[115]
**PAM/ gelatin/LiCl**	Electrolyte for ion transport	/	10.4	8.3	0.4	[116]
**PVA/HCl**	Electrolyte for ion transport	0.458	38.2	1.887	/	[117]
**PEDOT: PSS/MH**	Electrolyte for electron and ion transport	/	0.022	54,700	0.0014	[118]
**PEO/IL**	Electrolyte for ion transport	0.11	−15	0.18	0.0375	[119]
**PVA/IL**	Electrolyte for ion transport	0.28	4.85	2.78	0.025	[120]
**NaCl–PMSC/CNT-PAM**	Electrolyte for ion transport/ stretchable electrode	/	17.1	2.68	/	[121]
**PVA/IL**	Electrolyte for ion transport	0.29	10	0.16	/	[122]
**PAAc/XG/Bi_2_Se_0.3_Te_2.7_**	Substrate for inorganic materials	/	−0.45	5	/	[123]
**PEDOT: PSS/IL/**	Electrolyte for electron and ion transport	/	0.023	30,500	0.0098	[124]
**PEO/IL**	Electrolyte for ion transport	0.35	13	0.3	0.097	[125]
**Li_2_SO4/PAM/CA**	Electrolyte for ion transport	0.5085	11.5	1.072	0.141	[126]
**PAA/LiCl**	Electrolyte for ion transport	0.5286	11.3	5.98	/	[127]
**PAM/CMC/H_2_SO_4_**	Electrolyte for ion transport	0.4551	40.6	3.92	11.31	[128]
**PVA/PEDOT: PSS/Te-NWs**	Substrate for inorganic materials	0.468	0.787	1.5	0.000681	[129]
**PEGDA/2-HEA/IL**	Electrolyte for ion transport	0.215	38.9	0.0376	/	[130]
**PAAM/PDA/CNT-COOH/PANI**	Electrolyte for ion transport	0.68	18.6	17.53	6.06	[131]
	**Thermogalvanic (redox)**					
**AAc NPs**	Electrolyte for redox reaction	0.64	6.1	0.039	0.23 × 10^−3^	[132]
**AAc-co-NIPAM/NPs**	Electrolyte for redox reaction	/	−9.5	2	0.48 × 10^−3^	[133]
**BC/ Fe (CN)_6_^3−/4−^**	Electrolyte for redox reaction	/	4.5	6.81	/	[134]
**PAM/ Fe (CN)_6_^3−/4−^**	Electrolyte for redox reaction	1.3	1.37	1.05	0.31	[135]
**PAAM/ Fe (CN)_6_^3−/4−^**	Electrolyte for redox reaction	/	4.5	9.1	2.22	[136]
**PVA/ Fe (CN)_6_^3−/4−^**	Electrolyte for redox reaction	0.473	6.5	2.6	0.156	[137]
**Gelatin/ Fe (CN)_6_^3−/4−^/I^−^/I^3−^**	Electrolyte for redox reaction	/	5.2	0.45	5.2	[138]
	**Mixed ionic–electronic/protonic**					
**MOF/PEDOT: PSS**	Electrolytes for holes and ions	/	16.2	0.03	7.6	[139]
**PEDOT/PAMPS**	Electrolytes for the transport of holes and ions	0.136	−25.1	15.9	9.94	[140]
**CNC-PEDOT: PSS/PVA**	Electrolyte for hole transport	0.4385	1.31	4.73	0.00812	[141]
**BQ/H_2_Q**	Electrolyte for ions and proton-coupled electron transport	0.5379	25.9	7.41	5	[142]
**PVA/ PEDOT: PSS-SO_4_^2−^/SO_3_^2−^/NaCl**	Electrolyte for redox reaction and ion transport	0.6	1.63	2.92	/	[143]

**Table 4:** Literature review of flexible thermoelectric generators.

Process	P- & N-Type Materials	Elastomer	∆T (K)	Output Power (mW)	Reference
**Sandwich, cutting**	N-type Bi_2_Se_0.3_Te_2.7_ (0.38 × 0.38 × 0.38 mm^3^) P-type Bi_0.5_Sb_1.5_Te_3_ (0.38 × 0.38 × 0.38 mm^3^)	PI (substrate)	50	4.9	[150]
**Deposit**	N-type Bi_2_Te_3_ (film) P-type Bi_2_Te_3_ (film)	OSTE, PI	75	3.5 × 10^−5^	[151]
**Print**	P-type Sb_2_Te_3_ (15 × 20 × 0.5 mm^3^) N-type Bi_2_Te_3_ (15 × 20 × 0.5 mm^3^)	PDMS	50	1.18 (8 pairs)	[152]
**Sandwich**	p-type Bi_0.5_Se_1.5_Te_3_ (0.64 × 0.6 × 0.6 mm^3^) n-type Bi_2_Se_0.3_Te_2.7_ (0.64 × 0.6 × 0.6 mm^3^)	EGaIn (interconnect between legs) PDMS (substrate)	1.6	0.029	[153]
**Print**	P-type Bi_0.5_Sb_1.5_Te_3_ (film) N-type Bi_2_Te_2.7_Se_0.3_ (film)	PET (substrate)	25	1.2 × 10^−5^	[154]

## Data Availability

No new data was created.
